# Unlocking the therapeutic potential of P2X7 receptor: a comprehensive review of its role in neurodegenerative disorders

**DOI:** 10.3389/fphar.2024.1450704

**Published:** 2024-07-30

**Authors:** Xiaoming Liu, Yiwen Li, Liting Huang, Yingyan Kuang, Xiaoxiong Wu, Xiangqiong Ma, Beibei Zhao, Jiao Lan

**Affiliations:** ^1^ Shenzhen Baoan District Hospital of Traditional Chinese Medicine, Shenzhen, China; ^2^ Henan Hospital of Integrated Chinese and Western Medicine, Zhengzhou, China

**Keywords:** P2X7R, neuroinflammation, neurodegeneration, microglia, ATP

## Abstract

The P2X7 receptor (P2X7R), an ATP-gated ion channel, has emerged as a crucial player in neuroinflammation and a promising therapeutic target for neurodegenerative disorders. This review explores the current understanding of P2X7R’s structure, activation, and physiological roles, focusing on its expression and function in microglial cells. The article examines the receptor’s involvement in calcium signaling, microglial activation, and polarization, as well as its role in the pathogenesis of Alzheimer’s disease, Parkinson’s disease, multiple sclerosis, and amyotrophic lateral sclerosis. The review highlights the complex nature of P2X7R signaling, discussing its potential neuroprotective and neurotoxic effects depending on the disease stage and context. It also addresses the development of P2X7R antagonists and their progress in clinical trials, identifying key research gaps and future perspectives for P2X7R-targeted therapy development. By providing a comprehensive overview of the current state of knowledge and future directions, this review serves as a valuable resource for researchers and clinicians interested in exploring the therapeutic potential of targeting P2X7R for the treatment of neurodegenerative disorders.

## Graphical Abstract

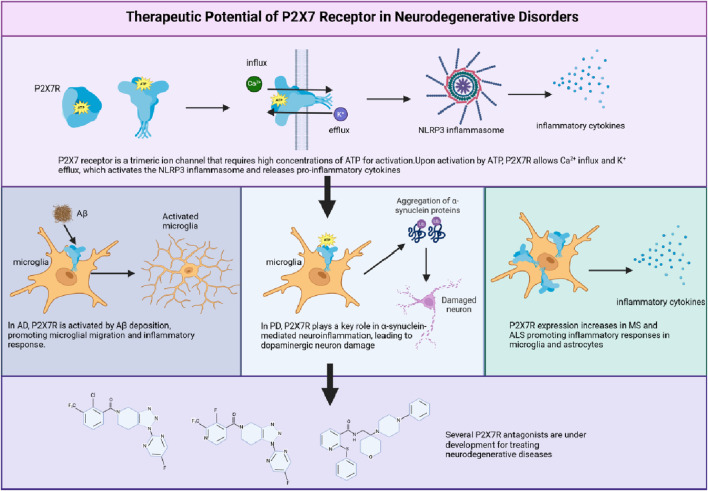

## 1 Introduction

The P2X7 receptor (P2X7R), an ATP-gated ion channel belonging to the P2X family, has recently emerged as a potential key player in the pathogenesis of various neurodegenerative disorders (Zheng et al., 2024). This receptor is predominantly expressed in immune cells, such as microglia, and regulates a wide range of cellular processes, including cell activation, calcium signaling, and cytokine release (Tewari et al., 2024). As a result, the P2X7R has been identified as a crucial mediator of neuroinflammation and immune responses within the central nervous system (CNS).

Growing evidence points to the P2X7R’s involvement in the onset and progression of neurodegenerative diseases, such as Alzheimer’s disease (AD) (Beltran-Lobo et al., 2023; Huang et al., 2024), Parkinson’s disease (PD) (Ren et al., 2021) and multiple sclerosis (MS) (Nobili et al., 2022), underscoring its potential as a therapeutic target. However, to fully harness the therapeutic potential of the P2X7R, a thorough understanding of its function, signaling pathways, and role in neurodegenerative disorders is crucial.

This review aims to provide a comprehensive overview of the current knowledge surrounding the P2X7R, with a focus on its expression in microglia, its role in calcium signaling, and its involvement in neurodegenerative disorders. We will explore recent advances in the development of P2X7R antagonists and their potential therapeutic applications, as well as discuss the challenges and future directions in P2X7R-targeted therapy research. The subsequent sections will delve into the P2X7R’s influence on microglial cell function through calcium signaling modulation, its role in neuroprotection and neurodegenerative disorders, and the current state of P2X7R antagonist research. Finally, we will synthesize the existing knowledge, identify research gaps and challenges, and provide future perspectives for the development of P2X7R-targeted therapies.

## 2 P2X7R

### 2.1 Unique characteristics of P2X7Rs

P2X7 receptors (P2X7Rs) exhibit unique properties that set them apart from other P2X receptors, making them an attractive target for research in various cellular contexts (Burnstock, 2017a). Activation of P2X7Rs necessitates higher ATP concentrations (−100 μM) compared to other P2X receptors (Surprenant et al., 1996). Upon activation, the P2X7R forms a non-selective cation channel, allowing the permeation of both monovalent (e.g., Na^+^ and K^+^) and divalent cations (e.g., Ca^2+^ and Mg^2+^) (North and Surprenant, 2000). Sustained activation can lead to the development of large, non-selective pores permeable to molecules up to 900 Da, a characteristic not observed in other P2X receptors (Pelegrin and Surprenant, 2006). The mechanism of P2X7R-mediated pore formation has been a subject of intense research and debate. Initially, it was thought that prolonged P2X7R activation led to the dilation of the receptor’s intrinsic channel, allowing the passage of larger molecules (Khakh and North, 2012). However, recent evidence suggests that the large pore formation may involve the recruitment and activation of additional proteins, such as pannexin-1 (Panx1) (Gulbransen et al., 2012). The P2X7R-Panx1 complex has been implicated in the release of ATP, glutamate, and other metabolites, as well as in the uptake of large molecules, such as fluorescent dyes (Gulbransen et al., 2012). However, the exact role of Panx1 in P2X7R-mediated pore formation remains controversial, as some studies have reported Panx1-independent mechanisms (Alberto et al., 2013). Moreover, recent structural studies have provided new insights into the potential mechanisms of P2X7R pore formation. The cryo-EM structure of the full-length rat P2X7R revealed a unique C-terminal domain that undergoes significant rearrangements upon ATP binding, potentially contributing to pore formation (McCarthy et al., 2019). Additionally, the identification of a cytoplasmic ballast domain and a C-cys anchor in the P2X7R structure suggests a role for these regions in the regulation of pore formation and channel gating (McCarthy et al., 2019). This distinctive feature is implicated in various physiological and pathological processes, such as the secretion of pro-inflammatory cytokines like interleukin-1β (IL-1β) and the induction of cell death (Di Virgilio et al., 2017).

### 2.2 Structure of P2X7Rs

P2X7Rs are trimeric ion channels expressed in various cell types throughout the body, particularly in macrophages and microglia (Collo et al., 1997). Each subunit is composed of 595 amino acids in mammals, forming a trimeric structure stabilized by hydrophobic and electrostatic interactions within its transmembrane domains and extracellular loop (Jiang et al., 2013; Karasawa and Kawate, 2016). Each subunit features two main structural domains: a cysteine-rich extracellular domain and the C- and N-termini, which enhance the channel’s functionality (Oken et al., 2022). The receptor includes extracellular, transmembrane, and cytoplasmic components (Tassetto et al., 2021). The ectodomain of P2X7R contains three ATP-binding sites and exhibits a chalice-like shape formed by interactions at various points (Jiang et al., 2013). Ligand-binding sites are created through interactions among the three extracellular domains of the subunits, which collectively comprise 282 amino acids (Di Virgilio et al., 2017; Jiang et al., 2021). Recent advancements in cryo-electron microscopy have shed light on the intricate structure of the full-length mammalian P2X7 receptor, revealing a more constricted channel in its apo state compared to the hP2X3 receptor, which effectively shields the orthosteric binding pocket from solvents and ligands (McCarthy et al., 2019). The transmembrane region of P2X7R consists of two domains: TM1 (amino acids 26–46) and TM2 (amino acids 330–349). TM1 harbors the ATP-binding pocket (Lara et al., 2020), while TM2 contains crucial pore-lining residues, such as S342 and Y343, which play a role in channel gating (Hansen et al., 1997; Pippel et al., 2017).

**FIGURE 1:**
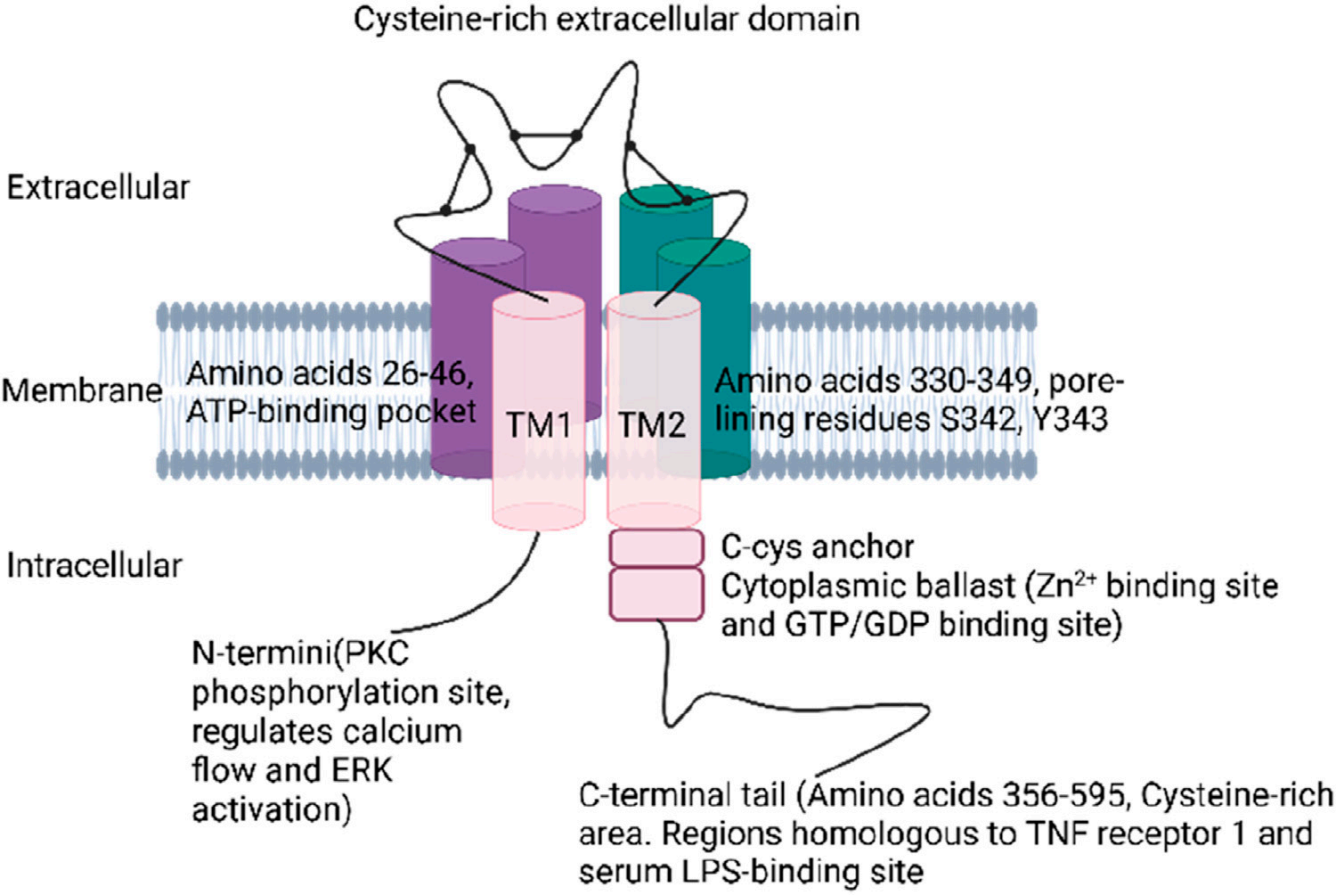
Structure of P2X7R.

The cytoplasmic region of P2X7R is characterized by distinctive elements, including the C-cys anchor and the cytoplasmic ballast. The C-cys anchor serves as a link between the transmembrane domain and the cytoplasmic cap, preventing receptor desensitization through multiple palmitoylated residues (McCarthy et al., 2019). The cytoplasmic ballast, on the other hand, features a novel fold that incorporates a dinuclear zinc ion complex and a high-affinity guanosine nucleotide-binding site, which likely facilitates the connection between the receptor and intracellular signaling proteins (Adinolfi et al., 2010). The N- and C-termini play crucial roles in receptor expression and function (Costa-Junior et al., 2011). The N-terminus, which begins approximately 26 amino acids earlier than other P2X receptors, contains a protein kinase C (PKC) phosphorylation consensus site and plays a role in regulating calcium flow, extracellular signal-regulated kinase activation, and P2X7R gating (Allsopp and Evans, 2011). The C-terminal tail, the most extensive among all P2XRs (amino acids 356–595), contributes to the stabilization of macropore opening and features a cysteine-rich area with palmitoylated residues that form a binding site for GDP/GTP and two zinc ions (Durner et al., 2023). The initial portion of the C-terminal tail contains a cysteine-rich region with palmitoylation on residues C362, C363, C374, C377, and S360, which function as hinges. Furthermore, the tail possesses regions that share homology with tumor necrosis factor receptor 1 and the serum LPS-binding protein, highlighting its multifaceted role in receptor function (Denlinger et al., 2001) (Figure 1).

### 2.3 Activation of P2X7R

**FIGURE 2:**
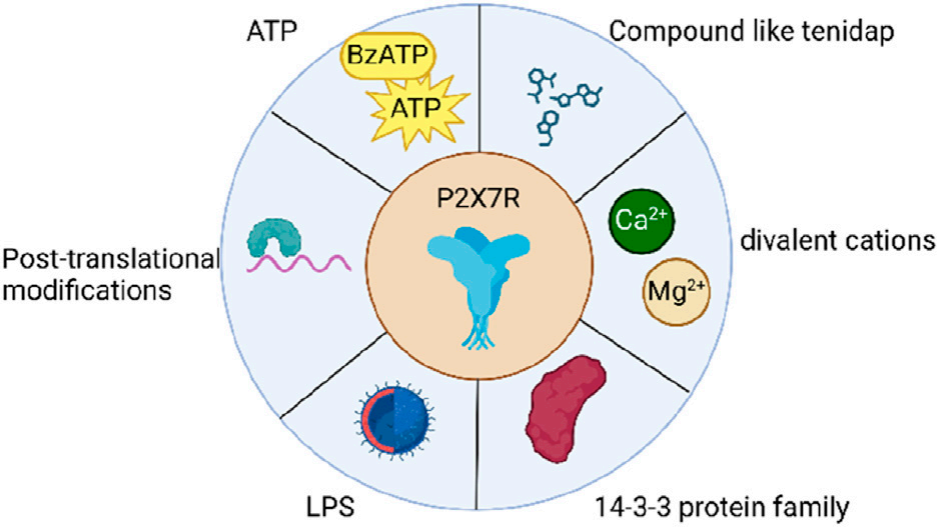
Items associated with the activation of P2X7R.

The activation of P2X7R is a complex process that involves the interplay of both extracellular and intracellular factors (Figure 2). Extracellular ATP serves as the primary ligand for P2X7R activation, with half maximal effective concentrations that vary across different species (Li et al., 2005; Bradley et al., 2011; He et al., 2017). Atomic modeling has revealed specific amino acid residues from two adjacent subunits that are crucial for ATP binding (Jiang et al., 2011; Hattori and Gouaux, 2012; Mansoor et al., 2016), while functional studies have highlighted the importance of surrounding residues in receptor activation (Stokes et al., 2006; Young et al., 2007; Adriouch et al., 2008; Liu et al., 2008; Martínez-Cuesta et al., 2020; De Salis et al., 2022). In addition to ATP, synthetic analogues such as BzATP and adenosine 5′-(γ-thio)-triphosphate can also activate P2X7Rs, albeit with varying potencies across species (Donnelly-Roberts et al., 2009). Divalent cations, including Ca^2+^ and Mg^2+^, act as allosteric inhibitors, modulating the activation of P2X7R (Roger et al., 2008). Intracellular regulation also plays a significant role in the activation of P2X7R. Protein-protein interactions, such as those with the 14-3-3 protein family, influence receptor trafficking and localization (Roger et al., 2008; Kopp et al., 2019). Post-translational modifications, including glycosylation, palmitoylation, and ubiquitination, alter the biophysical properties and stability of P2X7R, ultimately affecting its function and signaling (Oken et al., 2022; Li et al., 2023). Several compounds, such as.

LL-37, tenidap, polymyxin B, clemastine, ivermectin, and ginsenosides, have been identified as positive modulators of P2X7R activation (Elssner et al., 2004; Ferrari et al., 2006; Tomasinsig et al., 2008; Sanz et al., 2009; Nörenberg et al., 2011; 2012; Helliwell et al., 2015; Sivcev et al., 2019; Cui et al., 2023). LPS can modulate P2X7R activation through several distinct pathways. In murine cells, LPS stimulation of caspase-11 results in the cleavage of the ATP release channel pannexin-1, leading to an increase in extracellular ATP levels and subsequent P2X7R activation (Yang et al., 2015). Furthermore, intracellular LPS may reduce the threshold for P2X7R activation, possibly by interacting with an LPS-binding domain and inducing conformational changes that enhance the receptor’s sensitivity to ATP (Denlinger et al., 2001; Yang et al., 2015). However, further research is needed to provide direct evidence supporting this proposed mechanism. Moreover, Panx1 facilitates the release of ATP, which in turn activates P2X7R, resulting in caspase-3/7-mediated apoptosis and elevated IL-6 expression (Loureiro et al., 2022; Rusiecka et al., 2022). In summary, the activation of P2X7Rs is a multifaceted process influenced by various factors, including ligand binding, allosteric modulation, protein-protein interactions, post-translational modifications, and intracellular regulation.

### 2.4 Physiological roles of P2X7Rs

**FIGURE 3:**
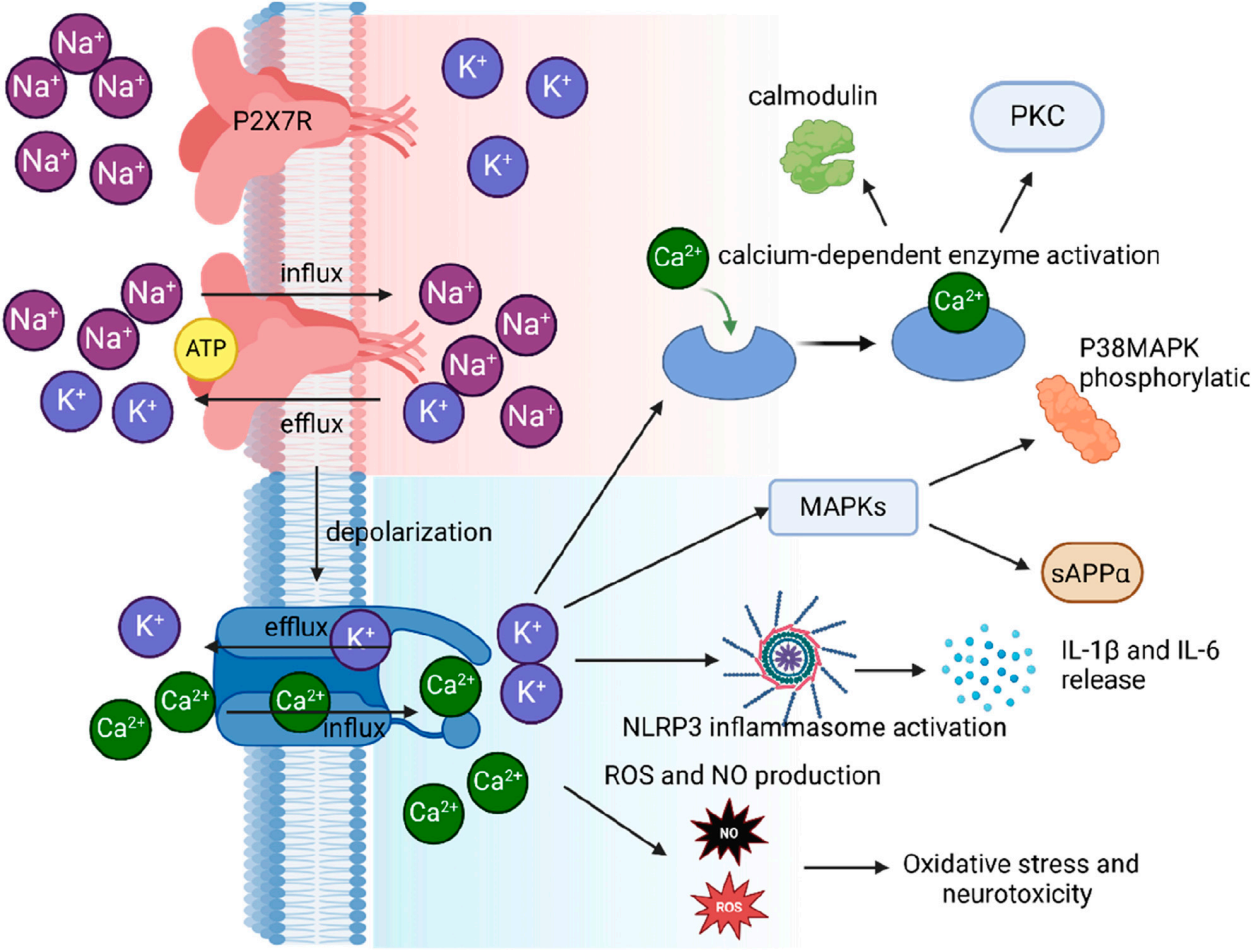
Illustration of P2X7R activation and its impact on ion flux, depolarization, and downstream signaling in microglial cells.

P2X7R participates in a wide array of physiological and pathophysiological processes, encompassing immune responses, inflammation, pain, and cell death (Ahn et al., 2023; Fan et al., 2024; Zou et al., 2024). In microglial cells, P2X7R activation leads to the release of pro-inflammatory cytokines, such as IL-1β and IL-18, through the activation of the NLRP3 inflammasome and caspase-1 (Xie et al., 2024), thereby contributing to the initiation and propagation of inflammatory responses in neurodegenerative diseases. Additionally, P2X7R plays a crucial role in regulating cell proliferation, differentiation, and migration (Di Virgilio et al., 2017). In certain cell types, such as osteoblasts and microglia, P2X7R activation can stimulate growth through increased intracellular calcium levels and the activation of downstream signaling pathways like MAPK and PI3K/Akt (Tóthová et al., 2021) (Figure 3). In contrast, P2X7R activation can inhibit proliferation in other cell types, such as lymphocytes, by triggering cell cycle arrest and apoptosis (Roger et al., 2008). P2X7R has been implicated in the differentiation of various cell types, including the promotion of osteoclastogenesis in bone remodeling (Gartland et al., 2012; Xu et al., 2023), modulation of neuronal differentiation in the CNS (Delarasse et al., 2009), and the glia proliferation in spinal cord (Stefanova et al., 2024). Moreover, P2X7R activation plays a crucial role in regulating the chemotactic response of immune cells, such as neutrophils, monocytes, and microglia, which is essential for immune surveillance and the resolution of inflammation (Chen et al., 2006; De Marchi et al., 2016). In conclusion, the P2X7R is a unique member of the P2X family with distinct properties and functions. Its complex structure and diverse physiological roles make it an attractive target for research in various cellular contexts.

## 3 The role of P2X7 receptor in calcium signaling and homeostasis in microglial cells

P2X7R plays a crucial role in modulating intracellular calcium levels, which in turn regulates a wide array of microglial physiological functions. This section will delve into the intricate interactions between the P2X7R and calcium channels, and how these interactions shape microglial behavior.

Upon activation by extracellular ATP, the P2X7R forms a cation-selective channel that permits the influx of Na^+^ and Ca^2+^ ions. While the rapid influx of Ca^2+^ through the P2X7R was once considered a critical event, with the receptor undergoing pore dilation under certain conditions (Di Virgilio et al., 2017), recent findings have challenged this traditional understanding. Peverini et al. questioned the concept of pore dilation, suggesting it might be an artifact of earlier research methodologies (Peverini et al., 2018). They emphasized the significance of lipid composition in the cell membrane, with lipids such as sphingomyelin and phosphatidylglycerol facilitating the passage of large cations through the P2X7R, while cholesterol hindered this process. Di Virgilio et al. unveiled the structural dynamics of the P2X7R, demonstrating that the N-terminus and transmembrane domains are crucial for the gating and dilation of the receptor, contrasting with the previous notion that the binding of three ATP molecules to the receptor was necessary for its activation (Karasawa and Kawate, 2016; Di Virgilio et al., 2018). Intracellular ions also contribute to the modulation of P2X7R activity, with intracellular magnesium ions inhibiting pore dilation (Harkat et al., 2017) and extracellular divalent cations influencing the conformational plasticity in the selectivity filter of the P2X7 pore (Khakh and North, 2006; Wang et al., 2020a), rather than altering the sensitivity of the P2X7R to ATP as previously thought.

Transient exposure to high concentrations of extracellular ATP (≥1 mM) or the more potent ATP analog, BzATP, elicits inward currents in human microglia with characteristics consistent with a P2X7R-mediated response. These currents are non-selective for cations, partially carried by Ca^2+^, enhanced during prolonged exposure to agonists, and inhibited by P2X7R antagonists (Janks et al., 2018). At the resting membrane potential of around −40 mV in rodent microglia (Nörenberg et al., 1994; Madry et al., 2018), eATP activation of P2X7Rs causes Na^+^ and Ca^2+^ to rush into the cell while K^+^ exits, resulting in membrane depolarization (Figure 3). This inward Ca^2+^ current increases the concentration of free intracellular Ca^2+^ ([Ca^2+^]i), triggering various cellular processes such as cell cycle progression (Bianco et al., 2006), TNF-α release (Hide et al., 2000), activation of the transcription factor NFAT(Ferrari et al., 1999), and disruption of the cytoskeleton (Mackenzie et al., 2005). The outward K^+^ current decreases intracellular K^+^ ([K^+^]i), activating the NLRP3 inflammasome and promoting the maturation and release of proinflammatory cytokines IL-1β and IL-18 (Di Virgilio, 2007) (Figure 3). While the inflammasome activates when [K^+^]i drops below 90 mM (Pétrilli et al., 2007), recent evidence suggests that the P2X7R is not the primary K^+^ efflux pathway (Swanson et al., 2019). Instead, the two-pore K^+^ channels, THIK-1 and TWIK-2, are responsible in microglia (Drinkall et al., 2022; Ossola et al., 2023)and macrophages (Di et al., 2018), respectively. Despite this, eATP promotes the recruitment and colocalization of microglial P2X7Rs and NLRP3 to discrete sites in the subplasmalemmal cytoplasm, suggesting that the inflammasome is positioned close enough to directly sense the P2X7R-mediated local drop in [K^+^]i (Franceschini et al., 2015).

Another mechanism by which the P2X7R can regulate intracellular Ca^2+^ levels in microglial cells is through the modulation of store-operated calcium entry (SOCE). SOCE is a major mechanism of calcium influx in non-excitable cells, and is activated upon the depletion of intracellular Ca^2+^ stores,

The diagram shows ATP binding to the P2X7R, which results in the influx of Na^+^ and Ca^2+^ ions and the efflux of K^+^ ions. This ion flux causes depolarization of the cell membrane, which subsequently leads to an increase in intracellular calcium levels. The elevated intracellular calcium triggers three distinct signaling pathways: 1) activation of calcium-dependent enzymes, including calmodulin and PKC; 2) activation of the NLRP3 inflammasome, leading to the release of IL-1β and IL-18; and 3) production of ROS and NO, contributing to oxidative stress and neurotoxicity. primarily from the endoplasmic reticulum (ER) (Kodakandla et al., 2023). SOCE is mediated by the interaction between the ER calcium sensor STIM1 and the plasma membrane calcium channel Orai1, which allows the influx of Ca^2+^ ions into the cell (Kodakandla et al., 2023). This P2X7R-induced SOCE activation can further amplify the calcium signaling in microglial cells, modulating various physiological functions such as cytokine production, phagocytosis, and migration (Kettenmann et al., 2013). Moreover, P2X7R-mediated calcium signaling can activate the mitochondrial apoptotic pathway, involving the release of cytochrome c and the activation of caspases, ultimately leading to cell death (Pelegrin and Surprenant, 2007).

The P2X7R can also modulate intracellular calcium homeostasis in microglial cells through its interaction with various calcium transporters and pumps. For example, the P2X7R activation can affect the activity of the plasma membrane Ca^2+^-ATPase (PMCA) and the sarco/endoplasmic reticulum Ca^2+^-ATPase (SERCA), which are responsible for maintaining calcium homeostasis by pumping Ca^2+^ ions out of the cytoplasm and into the ER, respectively (Recourt et al., 2020). It has been suggested that the P2X7R-mediated Ca^2+^ influx can lead to the activation of PMCA and SERCA, thereby restoring intracellular Ca^2+^ levels to their resting state and protecting the cell from Ca^2+^ overload and subsequent cell death (Di Virgilio et al., 2017). However, sustained activation of the P2X7R or exposure to high concentrations of ATP can lead to calcium overload and the induction of apoptosis (Pelegrin and Surprenant, 2006).

In conclusion, the P2X7R plays a central role in the regulation of calcium signaling and homeostasis in microglial cells, with profound implications for their physiological functions. The modulation of intracellular calcium levels by the P2X7R can influence various aspects of microglial cell function, including cytokine production, migration, phagocytosis, and cell survival. Given the importance of calcium signaling in the regulation of microglial functions, targeting the P2X7R and its interactions with calcium channels may provide novel therapeutic strategies for the treatment of neuroinflammatory and neurodegenerative disorders.

## 4 P2X7R in microglial activation and polarization

Microglia exhibit diverse activation states that extend beyond the conventionally defined M1 and M2 phenotypes (Gao et al., 2023). M1 microglia produce pro-inflammatory cytokines (TNF-α, IL-1β, IL-6) and express inducible nitric oxide synthase (iNOS), while M2 microglia promote tissue repair and anti-inflammatory responses by secreting IL-10, IL-4, and TGF-β. Transcriptomic analyses have revealed intermediate phenotypes, highlighting the complexity of microglial activation (Masuda et al., 2020). These cells dynamically interact with pro-inflammatory and phagocytic functions (Long et al., 2024). P2X7R critically regulates microglial activation, and its overexpression can increase membrane permeability, contribute to microgliosis, trigger inflammatory factor release, and induce neuronal damage (Zhang et al., 2020). Without an external ATP agonist, P2X7R enhances phagocytosis *via* interactions involving extracellular residues 306–320 and intracellular regions associated with non-muscle myosin heavy chain IIA (NMMHC IIA) and the cytoskeleton (Gu et al., 2009; 2010; 2011). The receptor’s closed state influences this phagocytic capacity, which can be disrupted by cytochalasin D or a monoclonal antibody targeting the extracellular P2X7R domain (Gu et al., 2011). P2X7R synthesis and surface expression are essential for human monocytic cell phagocytosis, and siRNA-mediated disruption impairs this function (Gu et al., 2011).

Transient extracellular ATP level increases cause P2X7R to transition from a scavenger receptor, promoting autophagy and driving microglia towards a mixed M1/M2 activation state (Gu and Wiley, 2018). The M1/M2 balance is crucial for microglia’s transformation into disease-associated microglia (DAM) in neurodegenerative diseases (Keren-Shaul et al., 2017). Activated P2X7R releases neurotransmitters into the CNS extracellular space (Illes et al., 2020) and could serve as a molecular target for PET imaging of M1 microglia (Lee et al., 2002; Parvathenani et al., 2003; Sanz et al., 2012). Upon activation, P2X7R can release neurotransmitters into the extracellular space in the CNS(Sperlágh et al., 2002). Studies also showed that P2X7R could serve as a potential molecular target for positron emission tomography (PET) imaging of M1 microglia (Territo et al., 2017; Tronel et al., 2017). P2X7R inhibition promotes the M2 phenotype, reducing neuroinflammation and improving functional recovery in a mouse spinal cord injury model (Chen et al., 2013). As such, P2X7R antagonists show potential in mitigating various neurodegenerative conditions (Beamer et al., 2017; Fabbrizio et al., 2017; Wang et al., 2017; Domercq and Matute, 2019; Illes et al., 2019). P2X7R antagonism effectively reduces neuroinflammation, plaque deposition, and cognitive deficits in AD animal models (Martin et al., 2019; Verkhratsky et al., 2019). Furthermore, P2X7R blockade limits microglial migration and activation, thereby reducing neurodegeneration in a PD mouse model (Alves et al., 2014).

Pathological persistent ATP stimulation activates P2X7R, triggering cellular responses that lead to NLRP3 inflammasome-mediated caspase-1 activation and pro-inflammatory cytokine secretion (Muñoz-Planillo et al., 2013; Illes et al., 2019) primarily stimulated by decreased intracellular potassium levels, highlighting P2X7R’s intricate ion regulatory role (Di Virgilio et al., 2017; 2018; Madry et al., 2018). P2X7R activation can also release other pro-inflammatory cytokines (IL-6, TNF-α), further exacerbating inflammation (Shieh et al., 2014).

**FIGURE 4:**
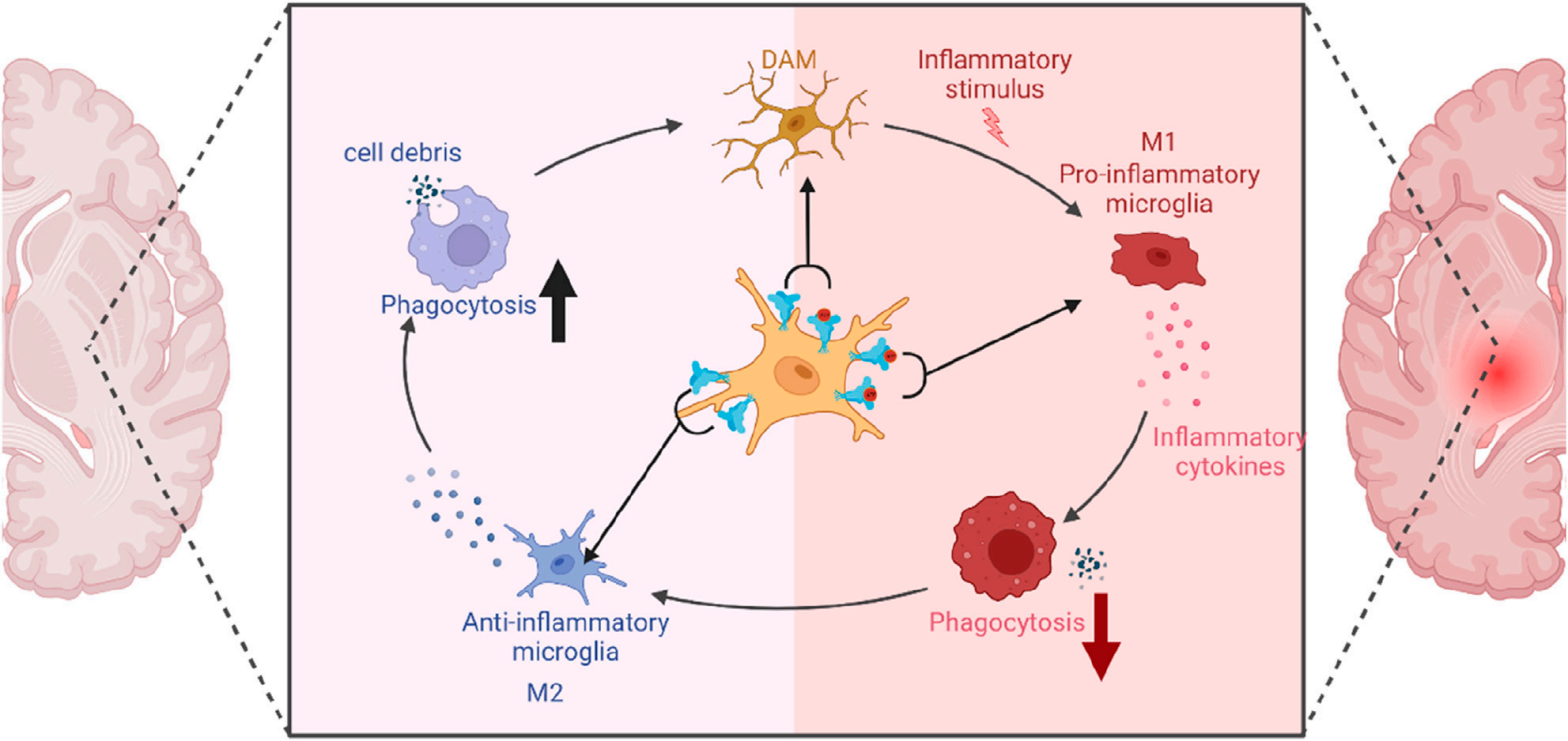
The role of P2X7R in microglial polarization.

In conclusion, P2X7R plays a pivotal role in coordinating pro-inflammatory and phagocytic responses, influencing microglial activation state diversity. Moreover, P2X7R is implicated in DAM state progression, particularly in neurodegenerative conditions like AD, by modulating the M1/M2 microglia activation balance (Figure 4).

This diagram illustrates the key role of P2X7R in shaping microglial polarization states and its influence on immune responses. Under normal conditions without redundant ATP, P2X7R is unactivated and induces phagocytic activity, resulting in minimal inflammation and predominance of the M2 state. However, transient ATP stimulation alters this balance: P2X7R not only drives phagocytosis but also gets activated by ATP, promoting inflammatory responses, thereby leading to a balance between M1 and M2 states (DAM). In a pathological condition, chronic ATP binding and activation of P2X7R shifts the cell primarily towards a pro-inflammatory and phagocytosis-suppressed phenotype, characteristic of the M1 state.

Considering the importance of P2X7R in microglial function and neuroinflammation, a deeper understanding of the molecular mechanisms governing its regulation and interaction with microglial activation states could pave the way for the development of targeted therapies. For instance, identifying the specific signaling pathways that mediate P2X7R-induced M1/M2 microglia polarization could enable the design of pharmacological agents that modulate these processes to prevent or slow down the progression of neurodegenerative diseases. Overall, the P2X7R emerges as a key player in microglial activation and function, with significant implications for the onset and progression of neurodegenerative diseases. As such, P2X7R presents an attractive target for the development of novel therapeutic strategies aimed at modulating neuroinflammation and ameliorating the symptoms of these debilitating conditions.

## 5 P2X7R related signaling pathway

### 5.1 MAPK

MAPKs, a family of serine/threonine kinases (p38MAPK, ERK, JNK), are pivotal regulators of gene expression, cellular differentiation, stress response, and cell survival or death in response to extracellular stimuli (Kim and Choi, 2010; Plotnikov et al., 2011). P2X7R activation is linked to MAPK phosphorylation, leading to various cellular outcomes. LPS-induced inflammation in microglia is mediated by p38, JNK, and ERK pathway activation, causing pro-inflammatory cytokine secretion (Lowe et al., 2012). BBG, a P2X7R inhibitor, attenuates this neuroinflammatory response by preventing MAPK phosphorylation and reducing cytokine production, an effect potentiated by MAPK inhibitors (Wang et al., 2020b). JNK and ERK contribute to TNF mRNA production, while p38 regulates its transport from the nucleus to the cytoplasm and stimulates its release from microglial cells in a P2X7R-dependent manner (Hide, 2003; Suzuki et al., 2004). The activation of JNK and p38 downstream of P2X7R involves SRC family kinases and appears independent of Ca^2+^ influx (Hide, 2003), although this relationship remains controversial (Jandy et al., 2022). Increased P2X7R expression in microglia and enhanced p38MAPK phosphorylation are associated with dopaminergic neuron loss in the nigrostriatal pathway in LPS-induced PD models (Herrera et al., 2000; Wang et al., 2017; Brown, 2019). BBG-mediated P2X7R inhibition reduces microglial activation and prevents p38MAPK-driven neurodegeneration (Wang et al., 2017). However, the effectiveness of P2X7R inhibition varies in different chemical PD models (Hracskó et al., 2011; Carmo et al., 2014), possibly due to differences in nigral damage extent or antagonist treatment duration.

Besides its role in neuroinflammation, P2X7R activation also modulates APP processing through MAPK-dependent mechanisms (Figure 3). P2X7R stimulation leads to the phosphorylation of ADAMs (ADAM9, −10, −17), facilitating the non-amyloidogenic a-processing of APP(Camden et al., 2005; Surprenant and North, 2009). An ADAM-independent process for APP a-processing has also been identified, involving Erk1/2 and JNK pathways, resulting in increased sAPPα release while suppressing sAPPβ and Aβ peptide production (Ma et al., 2005; Delarasse et al., 2011). Moreover, P2X7R signaling triggers ERM protein phosphorylation, which interact with P2X7R at the plasma membrane and promote APP processing and sAPPa shedding (Darmellah et al., 2012). P2X7R activation differentially modulates AKT and ERK pathways in a cell-type-specific manner. In rat cortical astrocytes, P2X7 activation induces AKT phosphorylation (Jacques-Silva et al., 2004), while in microglial cells, BzATP stimulation results in ERK and AKT dephosphorylation (Bian et al., 2013; He et al., 2017). Notably, P2X7R activation is not associated with IL-1 family cytokine release in this context (He et al., 2017).

In summary, the diverse roles of P2X7R in regulating MAPK signaling pathways underscore its significance in modulating neuroinflammation, neurodegenerative processes, and APP processing. Targeting P2X7R and its downstream effectors may offer novel therapeutic approaches for neurological disorders associated with aberrant MAPK activation. However, further studies are required to elucidate the complex interplay between P2X7R, MAPKs, and their cell-type-specific effects in the central nervous system.

### 5.2 ROS

Oxidative stress, characterized by elevated ROS levels and diminished antioxidant capacity, plays a crucial role in the development of neurodegenerative diseases (Orfali et al., 2024). Inflammation and metabolic stress disrupt mitochondrial function, which compromises energy production, leads to ROS accumulation, and causes neuronal death, accelerating disease progression (Millichap et al., 2021). A significant contributor to progressive neuronal death in these conditions is the inflammatory response due to microglial activation (Qin et al., 2023). This inflammatory response, along with P2X7R-mediated Ca^2+^ influx and mitochondrial dysfunction, exacerbates ATP-induced neurodegeneration and oxidative stress (Nishida et al., 2012; Jiang et al., 2015; Zelentsova et al., 2022). During ischemic and hypoxic conditions, mitochondria become a major ROS source by generating superoxide at the respiratory chain’s origin (Zelentsova et al., 2022). This superoxide can oxidatively modify and damage macromolecules within the neuronal plasma membrane (Subramaniam and Chesselet, 2013). The activation of P2X7R by α-synuclein decreases mitochondrial membrane potential, increases mitochondrial ROS production, and triggers the intrinsic apoptotic pathway, leading to mitochondrial dysfunction and cell death (Figure 3) (Wu and Bratton, 2013; Jiang et al., 2015; Wilkaniec et al., 2020). This process results in reduced energy production and promotes cell death through the activation of pro-apoptotic proteins (Wilkaniec et al., 2020).

NOX2, is a significant ROS producer in microglia, generating ROS as a byproduct of cellular metabolism and playing roles in both intracellular and extracellular signaling (Munoz et al., 2017). While extracellular ROS are harmful to neurons, intracellular ROS serve as signaling molecules that activate p38 and ERK1/2 in microglia, prompting the production of pro-inflammatory and neurotoxic cytokines (Zheng et al., 2024). Intriguingly, the absence of NOX2 in SOD1-G93A mice enhances disease progression and survival, suggesting a complex role for NOX2 in disease dynamics (Apolloni et al., 2013b). P2X7R stimulation by BzATP in SOD1-G93A microglia activates Rac1, a critical Rho GTPase family member, which in turn activates NOX1 and NOX2, leading to increased ROS production (Apolloni et al., 2013b; Munoz et al., 2017). Notably, P2X7R-mediated NOX2 activation and subsequent ROS generation in microglia are entirely dependent on Rac1 activation (Apolloni et al., 2013b). Furthermore, ATP stimulation of P2X7R in ALS microglia increases ERK1/2 phosphorylation, with a noted interdependence between the NOX2 and ERK1/2 pathways contributing to ROS overproduction (Lenertz et al., 2009; Apolloni et al., 2013b).

In spinal cord astrocytes, P2X7R activation results in ROS production and IL-6 release, partly mediated by NADPH oxidase (Munoz et al., 2020). In EOC13 cells, P2X7 activation leads to ROS formation and subsequent cell death (Hewinson and Mackenzie, 2007; Di Virgilio et al., 2009) through a mechanism independent of Ca^2+^ influx and K^+^ efflux (Bartlett et al., 2013), whereas in primary rat microglia, P2X7-induced ROS formation is dependent on Ca^2+^ influx (Kim et al., 2007).

### 5.3 NLRP3 inflammasome

The link between inflammation and neurodegenerative diseases is intricate, revealing a complex interplay that requires further study. Pro-inflammatory cytokines are widely associated with various neurodegenerative disorders (Allan and Rothwell, 2001), but their exact role in disease pathogenesis remains unclear. Although inflammation has been shown to contribute to disease progression in animal models, it is not conclusively identified as the primary initiator of neurodegeneration (Glass et al., 2010).

At the core of this inflammatory process is the NLRP3 inflammasome, a protein complex that includes the sensor protein NLRP3, the adaptor ASC, and the effector caspase-1 (Xu et al., 2020). Activation of P2X7R in microglia leads to the release of IL-1β and IL-18, driven by NLRP3 inflammasome activation and subsequent caspase-1 activation (Xie et al., 2024) (Figure 3). This activation occurs in two phases: initiation and activation (Bai et al., 2021). During the initiation phase, pattern recognition receptors (such as Toll-like receptors) detect PAMPs, DAMPs, or environmental stress, or NF-κB is activated by TNF-α, leading to increased expression of NLRP3, pro-IL-1β, and pro-IL-18 (Toma et al., 2010; Saha et al., 2020). ROS are crucial in this process, facilitating NF-κB activation and upregulation of inflammasome components (Zhang et al., 2018). The activation phase involves the assembly of NLRP3 and ASC with pro-caspase-1, converting it into active caspase-1. This activated enzyme cleaves pro-IL-1β and pro-IL-18, releasing mature cytokines that mediate inflammation (Stephenson et al., 2018). A variety of stimuli, including ATP, Nigericin, and alum, can trigger this inflammasome activation (Stephenson et al., 2018). Interestingly, in the mouse brain, microglia uniquely form functional NLRP3 inflammasomes, secrete IL-1β, and release IL-18 and IL-1α, while astrocytes do not exhibit this capability (Gustin et al., 2015). This specialization underlines the critical role of microglia in neuroinflammation and their involvement in neurodegenerative disease progression.

## 6 Antagonists targeting P2X7R

**FIGURE 5:**
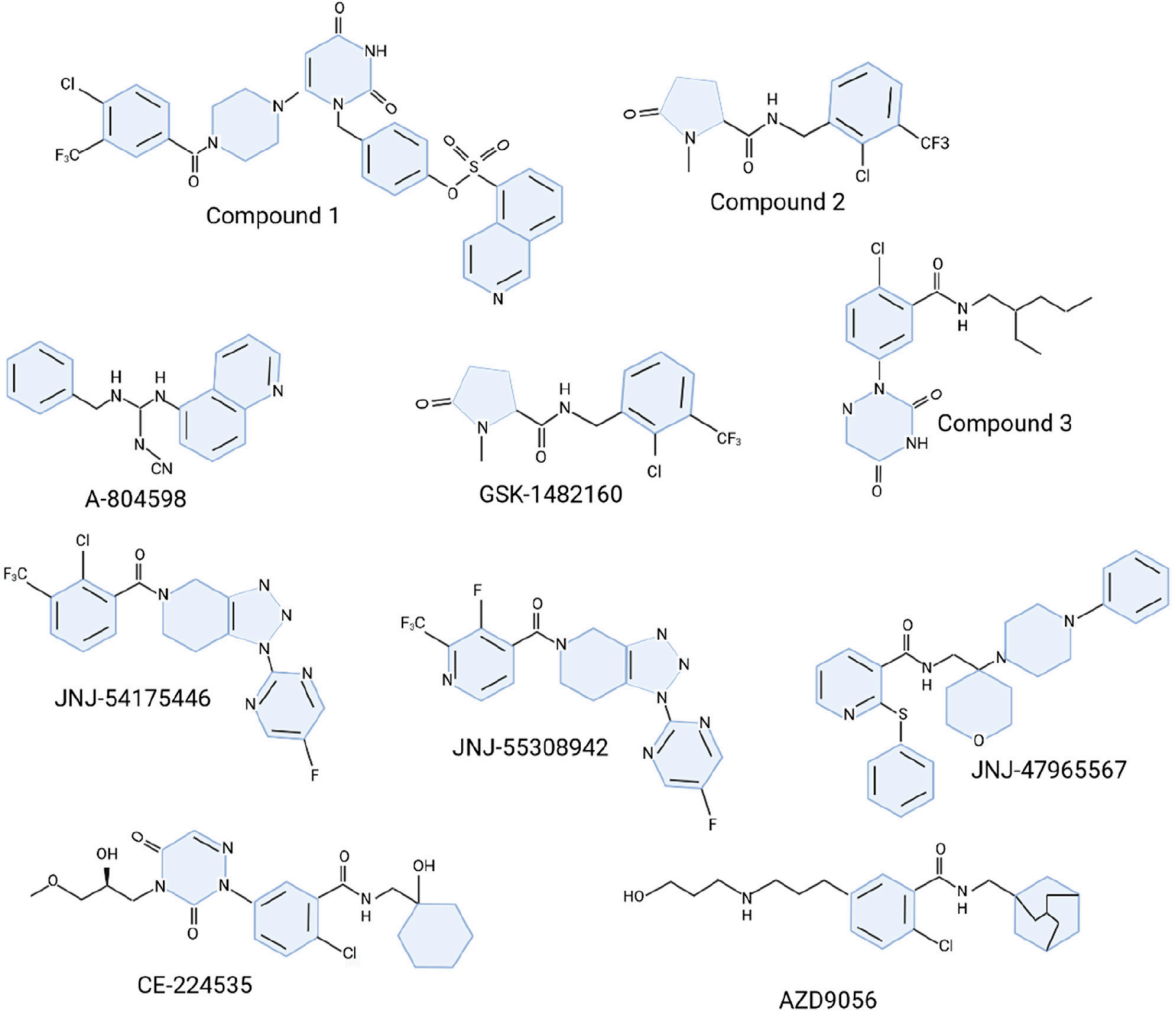
Molecular structure of P2X7R antagonists.

The P2X7R antagonists has made substantial advancements, although only a few compounds have entered clinical trials, predominantly for peripheral disorders (Stock et al., 2012; Chitnis and Weiner, 2017). The limited clinical progress is often due to challenges in understanding the human P2X7R (hP2X7R) structure and achieving drug-like properties suitable for central nervous system (CNS) targeting, such as optimal lipophilicity, solubility, and brain tissue half-life (Stock et al., 2012; Chitnis and Weiner, 2017). Initial development efforts involved nucleotide analogs with broad-spectrum antipurinergic activity, like oATP, which mitigates proinflammatory signaling through mechanisms independent of P2 receptors (Beigi et al., 2003; Huo et al., 2018). One notable antagonist, BBG, has been widely utilized in both *in vitro* and *in vivo* studies despite its selectivity and blood-brain barrier (BBB) permeability limitations (Gargett and Wiley, 1997; Keystone et al., 2012; Bin Dayel et al., 2019). The quest to develop P2X7R antagonists for CNS conditions has intensified, leading to the discovery of novel derivatives with improved pharmacological properties (Guile et al., 2009; Lee et al., 2009). High-throughput screening has identified promising chemical scaffolds, such as berberine alkaloid analogs and chiral pyrazolodiazepinones, which show effective hP2X7R blockade (Lee et al., 2009; Park et al., 2015). Pyrimidine-2,4-dione derivatives, including compound 1 (Figure 5), have demonstrated superior inhibition compared to earlier compounds like KN-62 (Chambers et al., 2010). Cyclic pyroglutamic amides, such as compound 2 (Figure 5), have shown enhanced P2X7R blockade and better metabolic stability (Abberley et al., 2010), while imidazole moieties like unsubstituted imidazolone 4 have been developed to increase half-life (Recourt et al., 2020).

The search for effective P2X7R antagonists has also explored other structures, including o-chlorobenzamide derivatives like compound 3 (Figure 5), which exhibited moderate inhibitory activity (Honore et al., 2006) (Figure 5). Cyanoguanidines such as A-804598 (Figure 5) have shown high potency and favorable pharmacokinetic (PK) profiles (Able et al., 2011). Adamantane derivatives and triazolopiperidine scaffolds have also been investigated, with compounds like JNJ-54175446 demonstrating promising PK properties and tolerability (Able et al., 2011; Savall et al., 2015; Swanson et al., 2016; Wilkinson et al., 2017). A new family of triazolopiperidines was developed, with JNJ-55308942 emerging as a candidate for clinical trials (Chrovian et al., 2018). Various heterocyclic derivatives were also explored by linking o-chlorobenzamide to quinoline (Chen et al., 2010; Letavic et al., 2013). Another ligand, JNJ-47965567, reduced amphetamine-induced locomotion sensitization but showed less efficacy in neuropathic pain and depression models (Bhattacharya et al., 2013).

Several P2X7R antagonists have reached clinical trials for conditions such as depression, rheumatoid arthritis (RA), and inflammatory bowel disease, but none have achieved market approval (Keystone et al., 2012; Ali et al., 2013; Eser et al., 2015). AstraZeneca’s AZD9056 and Pfizer’s CE-224535, developed for RA and osteoarthritis, were discontinued in phase II trials due to lack of efficacy (Keystone et al., 2012; Eser et al., 2015). Similarly, GlaxoSmithKline’s GSK-1482160, targeting chronic inflammatory pain, failed to achieve therapeutic benefits in phase I within the safe dose range (Ali et al., 2013).

Despite these setbacks, the ongoing development of P2X7R antagonists underscores the receptor’s potential as a therapeutic target for neuroinflammation and peripheral inflammatory disorders (Sperlágh and Illes, 2014; Zheng et al., 2017; Meyer et al., 2020). The focus has increasingly shifted towards compounds that can effectively penetrate the BBB, as seen with JNJ-54175446 and JNJ-55308942, offering hope for more targeted treatments for neuroinflammatory diseases (Bhattacharya et al., 2018; Recourt et al., 2020).

The pursuit of effective P2X7R antagonists for neuroinflammatory conditions represents a significant step forward in the development of novel therapies for these debilitating disorders. As our understanding of the role of P2X7R in neuroinflammation continues to expand, it is anticipated that more targeted and efficacious compounds will emerge, offering hope for patients suffering from these conditions. However, the path to successful drug development is not without challenges, as evidenced by the discontinuation of several P2X7R antagonists in clinical trials.

Despite the setbacks, the ongoing clinical development of several promising P2X7R antagonists highlights the potential of targeting this receptor for the treatment of various neuroinflammatory and peripheral inflammatory disorders. The following section will provide an in-depth look at the clinical progress and challenges associated with these P2X7R antagonists.

### 6.1 JNJ-54175446

JNJ-54175446 (Figure 5), a highly selective and potent P2X7R antagonist, marks a crucial advance in the development of brain-penetrant drugs for neuroinflammationJNJ-54175446 (Letavic et al., 2017). This compound exhibits impressive activity across different species with PIC50 values between 7.8 and 8.81. It boasts favorable physicochemical properties and efficient central nervous system partitioning. JNJ-54175446 demonstrates robust *in vivo* target binding following oral administration, with preclinical pharmacological studies showing a dose-dependent inhibition of IL-1β release in the rat hippocampus (Letavic et al., 2017). The liver microsome stability across species and its exceptional pharmacokinetic profile in rats, dogs, and non-human primates—characterized by low clearance rates (Cl = 2.9, 0.9, and 4.4 mL/min/kg, respectively), suitable intravenous half-lives (T1/2 = 8.6, 32, and 6.9 h, respectively), and high oral bioavailability (%F = 96, 164, and 157, respectively)—underscore its potential (Letavic et al., 2017). Preclinical success led to the advancement of JNJ-54175446 into clinical trials. In single-ascending dose studies with healthy volunteers, the compound was well-tolerated across doses ranging from 0.5 mg to 600 mg (Timmers et al., 2018). A subsequent clinical study assessed the drug’s efficacy in 312 subjects, including 69 patients with depression, who were administered daily doses of 50–150 mg. In this study, healthy participants were given 20 mg of D-amphetamine to evaluate mood responses, while depressed patients underwent total sleep deprivation. JNJ-54175446 significantly mitigated the mood effects of D-amphetamine in healthy subjects and attenuated mood elevation following sleep deprivation in depressed individuals (Lee et al., 2023). Further evaluation in a double-blind, placebo-controlled trial involved multiple doses of JNJ-54175446. The compound was well-tolerated across all tested doses and effectively reduced the locomotor effects of D-amphetamine, while enhancing mood-lifting effects at doses of 100 mg and higher (Recourt et al., 2020). The promising results from both preclinical and clinical studies suggest that targeting P2X7R with brain-penetrant antagonists like JNJ-54175446 could offer new avenues for treating neuroinflammatory conditions. Continued research and development in this area are essential as we deepen our understanding of neuroinflammation and the role of P2X7R in its progression, aiming to provide effective, targeted therapies for patients afflicted by these debilitating disorders.

### 6.2 JNJ-55308942

JNJ-55308942 (Figure 5), the second compound advanced into clinical trials, exhibited significant potency and selectivity across both human and rat models, with IC50 values of 10 nM for hP2X7R and 15 nM for rP2X7R (Chrovian et al., 2018). Unlike its predecessor JNJ-54175446, JNJ-55308942 demonstrated enhanced solubility and metabolic stability due to the substitution of isonicotinamides for benzamides, which also contributed to reduced CYP2C19 inhibition (Chrovian et al., 2018). This compound remained stable in rat and human liver microsomes and showed no significant hERG toxicity, with an IC50 exceeding 19 μM for various CYP450 isoforms.

Pharmacokinetic assessments indicated that JNJ-55308942 had poor to moderate clearance rates (Cl = 3.7, 0.8, and 2.6 mL/min/kg) and acceptable intravenous half-lives (T1/2 = 5.6, 21.2, and 6.0 h) in rats, dogs, and monkeys, respectively [178]. The compound also exhibited high exposure (AUC24h = 17,549, 23,262, and 16,262 (ng/mL)·h) and favorable oral bioavailability in these species (%F = 81, 98, and 97), suggesting efficient absorption [178]. Intravenous to oral dose ratios were 1/5 for rats, 0.25/1.25 for dogs, and 0.5/2.5 for monkeys. Estimated human pharmacokinetics predicted a clearance of 0.8 mL/min/kg and a volume of distribution at steady state (Vss) of 1.1 L/kg, with a projected half-life (T1/2) of 16 h.

Preclinical pharmacodynamic studies using an *in vivo* microdialysis assay demonstrated that oral administration of 10 mg/kg JNJ-55308942 significantly inhibited Bz-ATP-induced IL-1β release in the rat hippocampus at 1 and 5 h (Chrovian et al., 2018). In the *Bacillus* Calmette-Guerin (BCG)-induced depression model, the compound reversed deficits in sucrose preference and social interaction at a dose of 30 mg/kg (p.o.), indicating its efficacy in this model of depression (Bhattacharya et al., 2018). Additionally, in a rat chronic stress model, JNJ-55308942 reversed stress-induced deficits in sucrose intake, with autoradiography confirming increased P2X7R occupancy in the brain following treatment (Bhattacharya et al., 2018). Phase I clinical trials of JNJ-55308942 (NCT03151486) were conducted to assess its safety, tolerability, and pharmacokinetics in healthy male and female subjects. Although detailed results have not been disclosed, these trials are crucial for understanding the clinical potential of JNJ-55308942 (Chrovian et al., 2018).

### 6.3 SGM-1019

SGM-1019, an orally bioavailable small molecule P2X7R antagonist developed by Evotec, has shown potential in treating nonalcoholic steatohepatitis (NASH) (Dibba et al., 2018). With a Ki value of 7.6 nM for human P2X7R, SGM-1019 exhibited low efficacy in lower species, prompting relevant preclinical studies in non-human primate models (Dibba et al., 2018). These studies revealed that SGM-1019 significantly improved liver histology in CCL4-induced hepatic fibrosis models by decreasing stellate cell activation and alpha smooth muscle actin expression, reducing liver pathology, and improving overall histological scores (Baeza-Raja et al., 2020). Additionally, non-human primates treated with SGM-1019 demonstrated reduced hepatic stellate cell activation, decreased collagen deposition, and downregulation of fibrosis-related genes, along with lower serum alanine aminotransferase levels (Baeza-Raja et al., 2020). The promising preclinical outcomes in non-human primate models suggest that P2X7R antagonists like SGM-1019 have considerable antifibrotic potential *in vivo*. These findings laid the groundwork for exploring their therapeutic effects in clinical settings. A randomized, double-blind, placebo-controlled clinical trial (NCT03676231) involving 100 NASH patients was initiated to assess the preliminary safety, tolerance, pharmacokinetics, and efficacy of SGM-1019. The study involved twice-daily oral administration of the compound. However, the trial was prematurely terminated due to safety concerns (Dibba et al., 2018). Despite the early termination of the clinical trial, the preclinical evidence supporting the antifibrotic effects of P2X7R antagonists remains compelling. SGM-1019s ability to reduce liver pathology, improve histological scores, and modulate key fibrotic processes such as stellate cell activation and collagen deposition underscores its potential as a novel therapeutic agent for NASH (Baeza-Raja et al., 2020). This experience underscores the complexities of translating preclinical success into clinical efficacy and serves as a valuable learning opportunity for future drug development efforts.

### 6.4 GSK-1482160

GSK-1482160 (Figure 5), a pyroglutamic acid amide P2X7R antagonist, displays distinct affinities between human and rat receptors, with a higher affinity for human P2X7R (hP2X7R pIC_50_ = 8.5) compared to rat P2X7R (rP2X7R pIC_50_ = 6.5) (Abdi et al., 2010). The compound exhibits stability in liver microsome metabolism, showing clearance rates below 0.5 mL/min/g in both human and rat liver microsomes. Additionally, it does not significantly inhibit various CYP450 enzyme subtypes at concentrations up to 100 μM(Abdi et al., 2010).

Pharmacokinetic evaluations in rats demonstrated that intravenous administration of GSK-1482160 at 1 mg/kg resulted in low plasma clearance (9 mL/min/kg) and a half-life of 1.5 h. Oral administration at 3 mg/kg showed rapid absorption (Tmax = 1 h), an AUC of 73 min kg/L, and an oral bioavailability of 65% (Abdi et al., 2010). Efficacy studies revealed that GSK-1482160 effectively reversed mechanical allodynia in chronic joint pain and chronic constriction injury (CCI) models of neuropathic pain on the first day of treatment at 20 mg/kg, maintaining this effect throughout the treatment period (Abdi et al., 2010).

In clinical trials involving healthy volunteers, GSK-1482160 was tested for its pharmacokinetics and pharmacodynamics across doses ranging from 0.3 to 1,000 mg. It was well-tolerated and exhibited a good safety profile. However, pharmacokinetic/pharmacodynamic (PK/PD) modeling suggested that the compound could not achieve the pharmacological inhibition required for a full P2X7R mechanism of action (>90% IL-1β release inhibition) while maintaining a safe margin relative to the steady-state no-observed-adverse-effect level (NOAEL) (Ali et al., 2013). As a result, GSK-1482160 was deemed unsuitable for treating rheumatoid arthritis or other inflammatory diseases.

Despite its limitations as a therapeutic agent, GSK-1482160 has been successfully repurposed as a radioactive tracer for P2X7R imaging. When labeled with 11C or 18F, it serves as a novel radioligand due to its good central nervous system permeability, strong selectivity, and affinity for P2X7R (Han et al., 2017; Huang et al., 2022). This repurposing underscores the versatility of GSK-1482160 and the importance of considering species-specific differences in receptor affinity when translating preclinical findings to human applications.

### 6.5 CE-224535

CE-224535 (Figure 5), an azazyrimidine-like hP2X7R antagonist developed by Pfizer, shows strong activity against the receptor with Yo-Pro IC_50_ of 4 nM and IL-1β IC_50_ of 1.4 nM, but its efficacy in rodent models is limited, resulting in a lack of useful published data [196]. Pharmacokinetic studies across various animal species revealed significant differences. In rats, oral administration of CE-224535 (5 mg/kg) led to a plasma clearance rate of 11 mL/min/kg and a half-life of 2.4 h, but the oral bioavailability was low (%F = 2.6) [196]. Conversely, in monkeys and dogs, the half-life was shorter (T1/2 = 0.46 and 0.77 h, respectively), but oral bioavailability was significantly higher (%F = 59 and 22, respectively) (Duplantier et al., 2011). Daily administration of CE-224535 at 500 mg/kg (p.o.) for 4 days did not result in toxicity in rats, and the compound showed no significant effect on hERG at 1 μM or on major human CYP enzymes at 30 μM (Duplantier et al., 2011). These findings led to a 4-week phase I clinical trial (NCT00446784) to evaluate its safety and tolerability in 20 RA subjects already receiving methotrexate. However, results were not posted. This was followed by a 2-week, randomized, double-blind, placebo- and positive-controlled phase II trial (NCT00418782) to assess its analgesic and anti-inflammatory efficacy in osteoarthritis (OA) knee pain. This trial was terminated due to lack of efficacy, reflecting the challenges in translating potent *in vitro* activity into effective clinical outcomes (Stock et al., 2012). Subsequent efforts included a phase IIA study where CE-224535, administered at 500 mg twice daily for 12 weeks, failed to show efficacy in RA patients who did not respond adequately to methotrexate (Stock et al., 2012). The compound’s low efficacy in rodent models and suboptimal pharmacokinetic properties in some species might have contributed to its clinical shortcomings.

The development trajectory of CE-224535 underscores the difficulties of achieving therapeutic success with P2X7R antagonists, despite strong receptor affinity *in vitro*. The lack of significant clinical benefits in patients with advanced RA or those unresponsive to methotrexate suggests that targeting P2X7R alone may not suffice for effective treatment. This emphasizes the necessity of further research to understand the role of P2X7R within the context of existing treatment regimens and disease progression.

### 6.6 AZD9056

AZD9056 (Figure 5), a methyleneamide hP2X7R antagonist with an adamantane functional group, exhibits dose-dependent inhibition of BzATP-induced IL-1β and IL-18 release from human peripheral blood monocytes (IL-1β IC_50_ = 10–13 nM)(McInnes et al., 2014). Given its lack of rodent activity, its analogue AZ11657312 was tested in a rat streptococcal cell wall (SCW) arthritis model. AZ11657312, at doses of 30 and 60 mg/kg, reduced synovitis, sub-lining inflammation, chondronecrosis, and sub-chondral bone resorption in granulocytic precursors, suggesting potential as a novel RA treatment (McInnes et al., 2014).

In phase I trials, AZD9056 was well-tolerated in healthy participants, with a daily oral dose of 400 mg for up to 6 months, showing a good safety profile (Snell, 2014). The compound advanced to phase II clinical trials for RA, where phase IIa results indicated a reduction in swollen and tender joint counts compared to placebo. However, there was no significant effect on the acute phase response (Keystone et al., 2012). In a subsequent phase IIb study, AZD9056 failed to show any clinically or statistically significant efficacy in RA patients despite being well-tolerated [166]. The compound was also evaluated in a 28-day phase IIa trial for Crohn’s disease (CD). Here, the Crohn’s Disease Activity Index (CDAI) decreased from a mean of 311 to 242 in the AZD9056 group, compared to a decrease from 262 to 239 in the placebo group (*P* = 0.049). Despite these results, the study showed no changes in inflammatory biomarkers (C-reactive protein and fecal calprotectin), and the development for the CD indication was halted due to this discrepancy.AZD9056 was further assessed in a 4-week study for chronic obstructive pulmonary disease (COPD), where patients received 400 mg orally once daily. This study did not demonstrate any clinical benefits of the compound (Genetzakis et al., 2022).

The journey of AZD9056 underscores the challenges in translating preclinical efficacy into clinical success. Despite its potent inhibition of pro-inflammatory cytokines in human cells and promising results in animal models using its analogue, the compound did not achieve significant clinical efficacy in RA and CD patients (Keystone et al., 2012; McInnes et al., 2014). The discrepancy between clinical symptom improvement and the lack of biomarker changes in CD raises questions about the true impact on disease pathology (Eser et al., 2015). Additionally, the failure to show benefit in COPD patients highlights the complexity of P2X7R targeting across different diseases and underscores the necessity of a thorough understanding of its role in disease-specific contexts (Snell, 2014).

## 7 Involvement in neurodegeneration disorders

### 7.1 Alzheimer’s disease

AD is marked by the presence of neuronal fibrillary tangles and senile plaques, with hyperphosphorylated tau protein forming the tangles inside neurons and extracellular amyloid-beta (Aβ) peptides contributing to senile plaques (Martin et al., 2019).The production of Aβ involves cleavage of amyloid precursor protein (APP) by α-, β-, and γ-secretases, with P2X7R potentially influencing this process (Allinson et al., 2003). Specifically, P2X7R activation stimulates ADAM9, ADAM10, and ADAM17, which possess α-secretase activity and promote the non-amyloidogenic processing of APP(Camden et al., 2005; Surprenant and North, 2009). Additionally, P2X7R activates an alternative α-cleavage pathway independent of ADAMs, enhancing sAPPα release while reducing sAPPβ and Aβ peptide production (Delarasse et al., 2011). This P2X7R-mediated sAPPα release has been observed in various cell types, and P2X7R inhibition can prevent this effect.

Emerging evidence supports the role of P2X7R inhibition in restoring hippocampal synaptic integrity and plasticity, reducing Aβ pathology, and rescuing memory deficits in APPPS1 mice, without involving the sAPPα pathway, IL-1β treatment, microglial activation, or phagocytosis (Bhattacharya and Biber, 2016). Extracellular Aβ peptides (Sanz et al., 2009), released from damaged neurons and glial cells, activate P2X7R, which is upregulated during microgliocytosis and significant cognitive and motor impairment (Mucke et al., 2000). Elevated P2X7R expression correlates with AD progression, promoting microglia migration towards plaques and inhibiting phagocytosis (Martínez-Frailes et al., 2019). In familial AD (FAD), Aβ aggregation occurs before P2X7R expression in microglia, as demonstrated in the P2X7R-EGFP/J20 transgenic mouse model (Grad et al., 2017). Furthermore, P2X7R is implicated in the production of Aβ-induced chemokines and the recruitment of CD8^+^ T cells in the brain (Cartier et al., 2005). These chemokines, overexpressed in response to Aβ peptides *in vitro* and in AD models, lead to inflammation, immune cell recruitment, and neurodegeneration (Jaerve and Müller, 2012).Astrocytes are increasingly recognized for their roles in synaptic transmission and protein clearance pathways in AD, affecting both excitatory and inhibitory neurotransmission (Sung and Jimenez-Sanchez, 2020; Andersen et al., 2022). Dysregulation of calcium homeostasis by Aβ in astrocytes can impair neurotransmission modulation, leading to neuronal hyperactivity through mechanisms such as TRPA1 activation in APP/PS1 mice (Haughey and Mattson, 2003; Chow et al., 2010; Paumier et al., 2022).

P2X7R is a key modulator of astrocyte function in AD, influencing neurotransmission, synaptic dysfunction, oxidative stress, and neuroinflammation (Fellin et al., 2006; Lee et al., 2011; Diaz-Hernandez et al., 2012; Ficker et al., 2014; Martin et al., 2019; Ruan et al., 2020; Carvalho et al., 2021; Di Lauro et al., 2022). Recent studies suggest astrocytes produce L-serine, which is transported to neurons for D-serine production, critical for NMDA receptor-mediated synaptic plasticity. Impaired glucose metabolism in prodromal AD decreases L-serine synthesis, affecting cognitive function (Le Douce et al., 2020). Astrocytic expression of glutamate transporters, particularly EAAT2, near Aβ plaques remains debated (Hefendehl et al., 2016; Kobayashi et al., 2018). In APP/PS1 mice, lower EAAT2 levels around plaques lead to extrasynaptic glutamate accumulation and neuronal hyperactivity (Hefendehl et al., 2016), though human AD brain analyses suggest astrocytic resilience (Kobayashi et al., 2018).

Astrocytes also internalize modified tau proteins through mechanisms involving heparan sulfate proteoglycans (HSPGs) and LRP1, with uptake efficiency varying based on tau modifications (Martini-Stoica et al., 2018; Perea et al., 2019; Puangmalai et al., 2020; Rauch et al., 2020; Reid et al., 2020; Wang and Ye, 2021). Tau internalization and release might be mediated by HSPGs, and P2X7R regulates HSPG expression in other tissues, suggesting potential parallels in astrocytes (Mankus et al., 2012; Holmes et al., 2013; Merezhko et al., 2018). The role of P2X7R in regulating astrocytic HSPGs and tau clearance warrants investigation, particularly as astrocytes play a role in protein clearance in AD. P2X7R-induced regulation of astrocytic autophagy may limit the spread of pathological proteins in AD and tauopathies (Takenouchi et al., 2009b; 2009a; Kim et al., 2018; Lee and Kim, 2020).

Genetic deletion or pharmacological inhibition of P2X7R has demonstrated benefits in AD and frontotemporal dementia (FTD) mouse models, improving cognitive, behavioral, and synaptic deficits (Diaz-Hernandez et al., 2012; Martin et al., 2019; Ruan et al., 2020; Carvalho et al., 2021; Di Lauro et al., 2022). Astrocytic P2X7R may contribute to elevated glutamate levels near plaques through Ca^2+^-dependent mechanisms (Fellin et al., 2006; Ficker et al., 2014), highlighting the need for further research on its specific impact on astrocytic functions in AD. The therapeutic potential of targeting P2X7R in AD lies in its ability to modulate synapse-related and protein clearance functions of astrocytes, presenting a promising avenue for developing effective interventions for neurodegenerative disorders (Kim et al., 2011; 2018; Ryu et al., 2011; Lee and Kim, 2020).

### 7.2 Parkinson’s disease

PD, the second most common neurodegenerative disorder globally, affects over 6 million individuals and is a leading cause of neurological disability (GBD, 2016 Neurology Collaborators, 2019). Characterized by motor symptoms such as bradykinesia, rigidity, tremor, and postural instability, PD is pathologically defined by α-synuclein-positive inclusions (Lewy bodies and Lewy neurites) and the degeneration of dopaminergic neurons in the substantia nigra pars compacta and striatal projections (Sorrentino and Giasson, 2020; Tan et al., 2020). Although most cases are idiopathic, a minority have genetic origins (Tufekci et al., 2011).

Immune system dysfunction has emerged as a critical factor in PD’s susceptibility and progression (Tan et al., 2020). *Postmortem* analyses have revealed increased reactive microglia and phagocytic activity in the brains of PD patients (Pasqualetti et al., 2015). Elevated levels of pro-inflammatory cytokines such as IL-1β and TNF-α in the cerebrospinal fluid of these patients further support this (McGeer et al., 1988; Mogi et al., 1994; Blum-Degen et al., 1995). These immune responses, once considered secondary, are now seen as potential primary contributors to the disease (Maatouk et al., 2019).

Microglial activation, driven by α-synuclein, plays a pivotal role in PD neuroinflammation. Alpha-synuclein binds to microglial NOX2, activating the NOX complex and inducing oxidative stress *in vivo* (Wang et al., 2016), and directly activates P2X7R without the need for exogenous ATP (Wilkaniec et al., 2017). P2X7R involvement in α-synuclein-mediated microglial activation leads to ATP and ROS release, damaging dopaminergic neurons and contributing to PD pathology (Jiang et al., 2015; Wilkinson et al., 2017; Dos-Santos-Pereira et al., 2018). This process also involves the release of excitotoxic glutamate from activated microglia, further exacerbating neuronal damage through ATP/glutamate secretion and ROS, which contribute to the destruction of dopaminergic neurons and PD development (Sperlágh et al., 2002; Simon et al., 2020).

Animal studies have shown that P2X7R antagonism by Brilliant Blue G (BBG) can alleviate PD symptoms and protect dopaminergic neurons (Ferrazoli et al., 2017; Kumar et al., 2017; Crabbé et al., 2019; Oliveira-Giacomelli et al., 2019). For instance, in an LPS-induced PD model in rats, BBG treatment reversed the reduction of tyrosine hydroxylase-immunoreactive (TH-ir) neurons in the substantia nigra by inhibiting the P38MAPK pathway, highlighting the neuroprotective effects of P2X7R antagonism (Wang et al., 2017). Increased P2X7R expression on microglia, leading to dopaminergic neuron loss, was observed following LPS injection into the substantia nigra (Wang et al., 2017). BBG treatment reduced both microglial activation and dopaminergic neuron loss, indicating that P2X7R contributes to the synthesis of pro-inflammatory cytokines involved in neuronal death (Dutta et al., 2008). In rat models with 6-OHDA-induced nigrostriatal damage, BBG treatment decreased apomorphine-induced rotational behavior, suggesting a reduction in motor deficits associated with PD (Ferrazoli et al., 2017). Further studies suggest that P2X7R-mediated neuronal swelling and necrosis might be related to substantia nigra striatal dysfunction in PD. Exposure of SN4741 neuronal cells, derived from transgenic mouse embryos, to high ATP concentrations led to rapid cell volume increase and subsequent necrotic changes such as nuclear swelling, DNA leakage, and cytoplasmic vacuole formation (Jun et al., 2007).

Recent evidence implicates P2X7Rs in PD pathogenesis, particularly regarding microglial activation, neuroinflammation, and dopaminergic neuron loss. ATP binding to P2X7R activates microglia and recruits peripheral immune cells through complex pathways, including calcium influx, NLRP3 inflammasome activation, cell death induction, tissue damage, and further ATP release (Illes et al., 2020). This ATP/P2X7R/apoptosis axis creates a positive feedback loop, perpetuating the neurodegenerative process in PD (Illes et al., 2020).

In conclusion, targeting P2X7R offers a promising therapeutic strategy for PD. Future research should focus on the precise mechanisms of P2X7R-mediated neurotoxicity and the potential of P2X7R modulators as new therapeutic agents for PD [244–262].

### 7.3 Multiple sclerosis

MS is a chronic neurological condition marked by autoimmune-driven demyelination and axonal damage in CNS (Dendrou et al., 2015; Domercq et al., 2019; Domercq and Matute, 2019). The role of P2X7R in MS has garnered attention for their contribution to disease progression through diverse mechanisms (Burnstock, 2017b; Domercq et al., 2019; Domercq and Matute, 2019). In the relapsing-remitting form of MS, autoreactive T and B cells infiltrate the CNS, intensifying the inflammatory response (Mahad et al., 2015). As MS progresses, the focus shifts from peripheral immune cells to CNS-resident cells like microglia and astrocytes, which continue to sustain inflammation (Dendrou et al., 2015; Mahad et al., 2015; Domercq et al., 2019; Domercq and Matute, 2019). Notably, microglia expressing P2X7R are found in greater numbers in post-mortem spinal cord samples from MS patients than in non-MS controls (Yiangou et al., 2006; Amadio et al., 2017).

P2X7R upregulation is evident in activated microglia during the early stages of experimental autoimmune encephalomyelitis (EAE), an animal model of MS(Grygorowicz et al., 2018). Treatment with the P2X7R antagonist BBG in EAE models alleviates symptoms and reduces pro-inflammatory cytokine levels, including IL-1β, IL-6, and TNF-α(Sharp et al., 2008). In astrocytes, P2X7R expression also increases during EAE, and BBG administration lessens astrogliosis and neurological symptoms (Grygorowicz et al., 2016). Furthermore, Panx-1 knockout mice, which exhibit reduced ATP release, experience delayed EAE onset and lower mortality rates, underscoring the link between diminished P2X7R signaling and improved outcomes (Lutz et al., 2013).

In addition to influencing immune responses, P2X7R activation directly affects oligodendrocytes—the cells responsible for myelin formation in the CNS. Activation of P2X7R leads to significant Ca^2+^ influx, potentially causing oligodendrocyte death and exacerbating demyelination (Matute et al., 2007). Increased P2X7R expression in oligodendrocytes is observed in optic nerve samples from MS patients compared to controls (Chen and Brosnan, 2006). P2X7R is also detected on the abluminal surface of brain microvessels, impacting BBB integrity during MS. BBG treatment in EAE rats helps preserve BBB integrity and mitigates clinical symptoms (Sharp et al., 2008; Grygorowicz et al., 2018).Genetic studies reveal that single nucleotide polymorphisms (SNPs) in the P2X7R gene are associated with MS risk. The loss-of-function SNP rs28360457 (Arg307Gln) is linked to a reduced risk of MS, while the gain-of-function SNP rs17525809 (Val76Ala) is associated with an increased risk (Miller et al., 2011; Oyanguren-Desez et al., 2011; Gu et al., 2015).

### 7.4 Amyotrophic lateral sclerosis

ALS is a fatal neurodegenerative disorder characterized by the progressive loss of motor neurons in the brainstem and spinal cord, which control voluntary movement (Ghasemi and Brown, 2018; Cieślak et al., 2019). Sporadic cases usually manifest around age 60, while inherited forms appear around age 50, with survival typically ranging from two to 4 years due to respiratory failure (Ly et al., 2020). ALS pathogenesis involves a complex interplay of mechanisms, including oxidative stress, neuroinflammation, mitochondrial dysfunction, protein misfolding, trophic growth factor deficiencies, impaired axonal conduction, and blood-brain barrier disruption (Cieślak et al., 2019).

The SOD1-G93A transgenic mouse model, which overexpresses a mutant form of superoxide dismutase 1 (SOD1), is commonly used to study ALS and displays clinical symptoms at 70–140 days of age (Ly et al., 2020). P2X7R activation plays a critical role in ALS by driving chronic microglial activation and contributing to motor neuron death (Ruiz-Ruiz et al., 2020). *Postmortem* spinal cord samples from ALS patients show increased P2X7R immunoreactivity, primarily in microglia/macrophages within affected regions (Yiangou et al., 2006). *In vitro*, stimulation of P2X7R in SOD1-G93A mouse-derived spinal cord microglia with Bz-ATP increases TNF-α and cyclooxygenase-2 levels, inducing toxicity in neuronal cell lines, an effect that BBG antagonism can mitigate (D’Ambrosi et al., 2009). Furthermore, Bz-ATP enhances NADPH oxidase-2 activity in SOD1-G93A microglia, generating reactive oxygen species (ROS), which is preventable through P2X7R knockout or BBG treatment (Apolloni et al., 2013b). SOD1-G93A astrocytes also contribute to P2X7R-mediated ROS production (Gandelman et al., 2010). Co-cultures of motoneurons with SOD1-G93A astrocytes show motoneuron death that can be prevented by using MnTBAB, BBG, or apyrase to degrade ATP (Gandelman et al., 2010).

Aggregated and soluble SOD1-G93A activate the NLRP3 inflammasome in primary mouse microglia, leading to IL-1β release, whereas NLRP3-deficient microglia do not show this response, indicating a specific role for NLRP3 in ALS neurotoxicity (Deora et al., 2020). Interestingly, short-term P2X7R activation in SOD1-G93A mouse microglia initiates autophagy and increases anti-inflammatory biomarkers (M2 microglia), whereas prolonged activation disrupts autophagic flux, shifting microglia towards a pro-inflammatory phenotype (M1 microglia) (Fabbrizio et al., 2017). Conflicting evidence exists regarding the role of P2X7R in ALS progression. Ablation of P2X7R in SOD1-G93A mice accelerates clinical onset and worsens disease progression, leading to increased astrogliosis, microgliosis, motoneuron loss, and pro-inflammatory markers (Apolloni et al., 2013a). Conversely, BBG treatment in the late pre-symptomatic phase enhances motor neuron survival, reduces microgliosis in the lumbar spinal cord, and lowers inflammatory markers such as NADPH-2 oxidase, IL-1β, and BDNF (Apolloni et al., 2014). This suggests that P2X7R activation might be beneficial in early disease stages but harmful later on. However, other studies report only mild effects with P2X7R antagonism in female SOD1-G93A mice using BBG, and no effect with the highly selective P2X7R antagonist JNJ-47965567, indicating the influence of factors such as antagonist type, dosage, administration method, disease phase, and mouse gender on treatment efficacy (Bartlett et al., 2017; Sluyter et al., 2017; Ly et al., 2020).

In summary, P2X7R plays a complex and multifaceted role in ALS pathogenesis. Its activation in spinal cord microglia and astrocytes leads to neuroinflammation and motor neuron death through mechanisms involving the NLRP3 inflammasome, ROS, and ATP production. The contrasting effects of P2X7R activation at different disease stages complicate its therapeutic potential. Further research should focus on understanding how P2X7R contributes to ALS pathology and refining the use of P2X7R modulators for effective treatment of ALS.

## 8 Research gaps and future perspectives for P2X7R-targeted therapy

The P2X7 receptor has emerged as a promising therapeutic target for various neurodegenerative disorders, including AD, PD, MS, and ALS. Despite the growing body of evidence supporting the involvement of P2X7R in the pathogenesis of these diseases and its potential as a therapeutic target, several research gaps and challenges need to be addressed to fully harness the therapeutic potential of P2X7R-targeted therapies.

One of the primary challenges is the complex and multifaceted role of P2X7R in neuroinflammation and neurodegeneration. While P2X7R activation is generally associated with pro-inflammatory responses and neurotoxicity, some studies have suggested that brief P2X7R stimulation may have neuroprotective effects, particularly in the early stages of disease progression. This dual function of P2X7R highlights the need for a more nuanced understanding of its role in different disease contexts and stages. Future research should focus on elucidating the precise mechanisms underlying the beneficial and detrimental effects of P2X7R activation, as well as identifying the optimal therapeutic window for P2X7R-targeted interventions.

Another important aspect is the characterization of P2X7R expression in different cell types within the central nervous system, particularly in neurons. While P2X7Rs are predominantly expressed in glial cells, such as microglia and astrocytes, their presence in neurons remains a topic of ongoing debate (Illes et al., 2017). Some studies have reported the expression of functional P2X7Rs in neurons, suggesting their involvement in neurotransmission, synaptic plasticity, and neuronal survival (Armstrong et al., 2002; Miras-Portugal et al., 2003). However, other studies have questioned the specificity of the antibodies and techniques used to detect neuronal P2X7Rs, leading to conflicting results (Anderson and Nedergaard, 2006; Illes et al., 2017). To resolve this controversy, future research should focus on developing highly specific tools for detecting P2X7R expression in neurons, such as novel antibodies, reporter mouse lines, or *in situ* hybridization techniques (Kaczmarek-Hajek et al., 2018). Additionally, investigating the functional role of neuronal P2X7Rs in different brain regions and under various physiological and pathological conditions will provide valuable insights into their potential involvement in neurodegenerative processes. Elucidating the cell-type specific expression and function of P2X7Rs will contribute to a better understanding of their therapeutic potential and guide the development of targeted interventions for neurodegenerative diseases.

To address these challenges, a comprehensive understanding of the molecular mechanisms underlying P2X7R signaling is crucial. While the P2X7R has been implicated in various cellular processes, including cell activation, calcium signaling, and cytokine release, the intricate molecular mechanisms underlying these effects remain elusive. Employing systems-level approaches, such as omics technologies and computational modeling, could shed light on these mechanisms and facilitate the development of more selective and effective P2X7R-targeted therapeutics.

Moreover, clarifying the context-specific role of P2X7R in different cell types and disease stages is essential. The P2X7R is predominantly expressed in immune cells like microglia and is also found in astrocytes. However, it is important to note that there is an ongoing debate regarding the existence of P2X7R in neurons (Illes et al., 2017; Miras-Portugal et al., 2017; Kaczmarek-Hajek et al., 2018). While some studies have reported the presence of P2X7R in neurons, others have questioned the specificity of the antibodies and techniques used to detect neuronal P2X7R expression. As a result, the extent of P2X7R expression and function in neurons remains a topic of active investigation. Nonetheless, a more nuanced understanding of the spatiotemporal dynamics of P2X7R expression and function across glial cell types and disease stages will help to determine its overall contribution to neurodegenerative disorders and inform the development of targeted therapies.

Another important aspect is the characterization of the functional diversity of P2X7R isoforms. Numerous P2X7R isoforms have been identified, yet their functional significance and involvement in disease progression remain poorly understood. Systematic functional analyses of distinct P2X7R isoforms could reveal novel therapeutic targets and provide insight into disease-specific mechanisms.

The development of brain-penetrant P2X7R antagonists remains a significant hurdle in the pursuit of effective therapies for neurodegenerative disorders. While some compounds, such as JNJ-54175446 and JNJ-55308942, have demonstrated improved brain penetrance and promising results in preclinical studies, further research is needed to optimize their pharmacokinetic and pharmacodynamic properties and assess their safety and efficacy in clinical trials. Moreover, efforts are needed to develop more selective and potent P2X7R modulators that can effectively penetrate the BBB and exhibit favorable pharmacological properties.

Addressing the translational gap in P2X7R-targeted therapies is another critical aspect. While preclinical studies have shown encouraging results, more extensive clinical trials are required to validate the long-term safety and efficacy of P2X7R-targeted therapies. Rigorous assessment of potential off-target effects, optimal dosing, treatment duration, and patient stratification strategies will be critical to the successful translation of these therapies to the clinic.

Finally, given the complex and multifactorial nature of neurodegenerative disorders, P2X7R-targeted therapies may need to be combined with other therapeutic approaches to achieve maximal clinical benefit. Systematic identification of synergistic combinations of P2X7R-targeted therapies with other treatments, such as immunomodulatory, neuroprotective, or neurorestorative agents, will be essential for optimizing their therapeutic potential. Exploring alternative strategies for modulating P2X7R function, such as targeting downstream effectors of P2X7R signaling or investigating the potential of gene therapy or RNA-based therapeutics, may also offer new avenues for therapeutic development.

In conclusion, while P2X7R-targeted therapy holds great promise for the treatment of neurodegenerative disorders, significant research gaps and challenges remain. Addressing these issues will require a concerted effort from the scientific community, involving a combination of basic research, preclinical studies, and well-designed clinical trials. By deciphering the complex molecular mechanisms of P2X7R signaling, clarifying its context-specific role in different cell types and disease stages, characterizing the functional diversity of isoforms, overcoming pharmacological challenges, addressing the translational gap, and unraveling the potential of combination therapies, we may pave the way for the development of more effective and targeted interventions for these devastating diseases. Future perspectives for P2X7R-targeted therapy development should focus on these critical aspects to maximize the therapeutic potential of this promising target.

## Figures and Tables

**Table:** 

**ADAMs**	A Disintegrin and Metalloproteinases
**AD**	Alzheimer’s Disease
**ALS**	Amyotrophic Lateral Sclerosis
**APP**	Amyloid Precursor Protein
**ATP**	Adenosine Triphosphate
**BBB**	Blood-Brain Barrier
**BBG**	Brilliant Blue G
**BCG**	*Bacillus* Calmette-Guerin
**BzATP**	3′-O-(4-Benzoylbenzoyl) Adenosine 5′-Triphosphate
**CCI**	Chronic Constriction Injury
**CD**	Crohn’s Disease
**CDAI**	Crohn’s Disease Activity Index
**CNS**	Central Nervous System
**COPD**	Chronic Obstructive Pulmonary Disease
**DAMPs**	Damage-Associated Molecular Patterns
**DAMs**	Disease-Associated Microglia
**EAE**	Experimental Autoimmune Encephalomyelitis
**ER**	Endoplasmic Reticulum
**ERK**	Extracellular Signal-Regulated Kinase
**FAD**	Familial Alzheimer’s Disease
**FTD**	Frontotemporal Dementia
**GDP**	Guanosine Diphosphate
**GTP**	Guanosine Triphosphate
**hP2X7R**	Human P2X7 Receptor
**HSPGs**	Heparan Sulfate Proteoglycans
**IL-1β**	Interleukin-1β
**iNOS**	Inducible Nitric Oxide Synthase
**JNK**	c-Jun N-terminal Kinase
**LPS**	Lipopolysaccharide
**MAPKs**	Mitogen-Activated Protein Kinases
**MS**	Multiple Sclerosis
**NASH**	Nonalcoholic Steatohepatitis
**NLR**	Nucleotide-Binding Oligomerization Domain-Like Receptor
**NLRP3**	NLR Family Pyrin Domain Containing 3
**NMMHC**	IIA Non-Muscle Myosin Heavy Chain IIA
**NOAEL**	No-Observed-Adverse-Effect Level
**NOX2**	NADPH Oxidase 2
**OA**	Osteoarthritis
**oATP**	Oxidized ATP
**PAMPs**	Pathogen-Associated Molecular Patterns
**Panx1**	pannexin-1
**PD**	Parkinson’s Disease
**PET**	Positron Emission Tomography
**PK**	Pharmacodynamic
**PKC**	Protein Kinase C
**PMCA**	Plasma Membrane Ca2+-ATPase
**rP2X7R**	Rat P2X7 Receptor
**RAD**	Rheumatoid Arthritis
**ROS**	Reactive Oxygen Species
**sAPPα**	Soluble Amyloid Precursor Protein-α
**SCW**	Streptococcal Cell Wall
**SERCA**	Sarco/Endoplasmic Reticulum Ca2+-ATPase
**SNPs**	Single Nucleotide Polymorphisms
**SOCE**	Store-Operated Calcium Entry
**SOD1**	Superoxide Dismutase 1
**Th17**	T Helper 17 Cells
**TNF-α**	Tumor Necrosis Factor-α
